# Neural-Network Based Modeling of I/O Buffer Predriver under Power/Ground Supply Voltage Variations

**DOI:** 10.3390/s21186074

**Published:** 2021-09-10

**Authors:** Malek Souilem, Jai Narayan Tripathi, Rui Melicio, Wael Dghais, Hamdi Belgacem, Eduardo M. G. Rodrigues

**Affiliations:** 1 École Nationale d’Ingénieurs de Sousse, Université de Sousse, Sousse 4054, Tunisia; malek.souilem@ua.pt; 2 Laboratoire d’Electroniques et Microélectroniques, Université de Monastir, Monastir 5000, Tunisia; belgacem.hamdi@gmail.com; 3 Department of Electrical Engineering, Indian Institute of Technology Jodhpur, Jodhpur 342037, India; jai@iitj.ac.in; 4 IDMEC, Instituto Superior Técnico, Universidade de Lisboa, 1049-001 Lisboa, Portugal; 5 ICT—Instituto de Ciências da Terra, Universidade de Évora, Rua Romão Ramalho 59, 7000-671 Évora, Portugal; 6 Institut Supérieur des Sciences Appliquées et de Technologie de Sousse, Université de Sousse, Sousse 4003, Tunisia; waeldghais@ua.pt; 7 Management and Production Technologies of Northern Aveiro—ESAN, Estrada do Cercal 449, Santiago de Riba-Ul, 3720-509 Oliveira de Azeméis, Portugal; emgrodrigues@ua.pt

**Keywords:** VLSI, I/O, behavioral modelling, IBIS, power supply induced jitter, nonlinear dynamic circuits, neural network, parametric modelling, system identification

## Abstract

This paper presents a neural-network based nonlinear behavioral modelling of I/O buffer that accounts for timing distortion introduced by nonlinear switching behavior of the predriver electrical circuit under power and ground supply voltage (PGSV) variations. Model structure and I/O device characterization along with extraction procedure were described. The last stage of the I/O buffer is modelled as nonlinear current-voltage (I-V) and capacitance voltage (C-V) functions capturing the nonlinear dynamic impedances of the pull-up and pull-down transistors. The mathematical model structure of the predriver was derived from the analysis of the large-signal electrical circuit switching behavior. Accordingly, a generic and surrogate multilayer neural network (NN) structure was considered in this work. Timing series data which reflects the nonlinear switching behavior of the multistage predriver’s circuit PGSV variations, were used to train the NN model. The proposed model was implemented in the time-domain solver and validated against the reference transistor level (TL) model and the state-of-the-art input-output buffer information specification (IBIS) behavioral model under different scenarios. The analysis of jitter was performed using the eye diagrams plotted at different metrics values.

## 1. Introduction

Signal and power integrity (SPI) simulation of high-speed mixed-signal I/O links is a fundamental task that designers perform and iterate until meeting the specification of timing and amplitude distortions. SPI involves the prediction of the impact of the supply voltage variations on the timing and amplitude distortions of the output signal propagating on package and PCB interconnects [1].

A behavioral model based on input-output buffer information specifications (IBIS) or other parametric and enhanced equivalent circuit approaches can be used in SPI simulation flow that balances the tradeoff between simulation time and computational resources with good accuracy [2,3]. Nevertheless, previous nonlinear behavioral modelling methodologies focus mainly on improving the modelling of the last-stage of the I/O buffer [4,5,6,7]. In fact, voltage-time (V-t) tables capturing the predriver’s I/O timing distortions are extracted under fixed predriver’s power and ground supply voltage (PGSV) $V_{dd}/V_{ss}$ DC voltage. For this reason, an equivalent circuit or parametric behavioral modelling, which are generated under the above V-t conditions, will not accurately predict the predriver’s output timing distortions, which are the input of the last-stage driver model. Moreover, this shortcoming limits the usage of the behavioral models when they are subjected to supply ripple voltage derived from frequency domain simulations [8,9,10,11,12].

**Figure 1:**
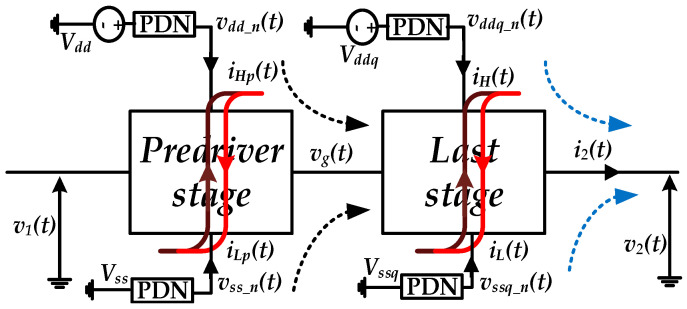
I/O buffer block diagram with separate supply domains for predriver and last stage independently impacting the output timing and amplitude distortion.

For instance, PGSV variations at the predriver and last stage would distort the timing and the amplitude $v_{g}\left(t\right)$ and $v_{2}\left(t\right)$, respectively, of the output voltage, as is illustrated in Figure 1. The arrows in Figure 1 highlight the nonlinear dynamic effects showed by the predriver and last stage since they are designed based on transistors. The black dashed arrows present the induced jitter by $v_{dd_{n}}\left(t\right)$ and $v_{ssd_{n}}\left(t\right)$ on $v_{g}\left(t\right),$ and the output voltage of the predriver and the blue dashed arrows present the induced jitter by $v_{ddq_{n}}\left(t\right)$ and $v_{ssq_{n}}\left(t\right)$ on $v_{2}\left(t\right)$, the output voltage of the driver.

**Figure 2:**
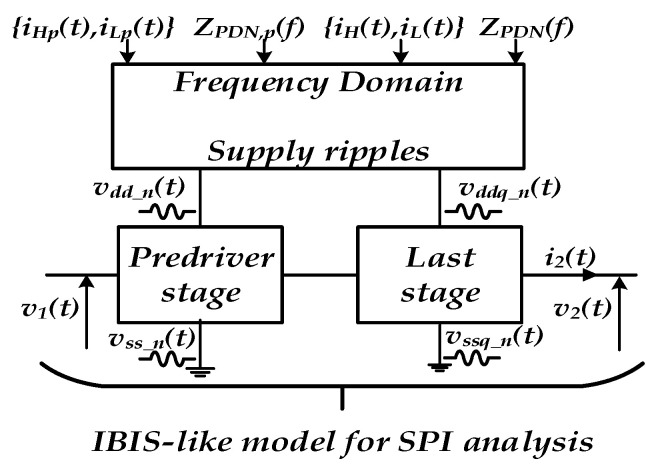
Combined flow for PGSIJ transient simulation based on the determination of PGSV ripple noise from the frequency domain analysis.

The extrinsic linear PDN network effects can be simulated in frequency domains, while the nonlinear distortion effects induced by the I/O buffer currents are simulated in time-domain analysis. Therefore, Figure 2 depicts the integrated transient simulation flow of PGSIJ based on the determination of supply ripple noise from frequency domain analysis.

By assuming that the switching current at the predriver, $\left\{i_{H,p}\left(t\right),\;i_{L,p}\left(t\right)\right\}$, and at the last stage level, $\left\{i_{H}\left(t\right),\;i_{L}\left(t\right)\right\}$, flow through the power delivery network (PDN) impedance, $Z_{PDN,p}$ and $Z_{PDN}$, supply ripple can be determined in the frequency domain: $v_{dd\_n}\left(f\right)=Z_{PDN,p}\left(f\right)\cdot \;i_{Hp}\left(f\right)$ and $v_{ddq\_n}\left(f\right)=Z_{PDN}\left(f\right)\cdot \;i_{H}\left(f\right)$. Then, the time-domain supply noise waveform can be determined via inverse fast Fourier transform (i.e., $FFT^{-1}$):

(1) $$\{\begin{array}{c} v_{dd\_n}\left(t\right)=FFT^{-1}\left[v_{dd\_n}\left(f\right)\right]\;\\ v_{ddq\_n}\left(t\right)=FFT^{-1}\left[v_{ddq\_n}\left(f\right)\right] \end{array}$$

Then, these voltages in (1) are injected to the I/O buffer behavioral model supply terminals at both predriver and last stage for predicting the SPI distortion of high-speed I/O links.

**Figure 3:**
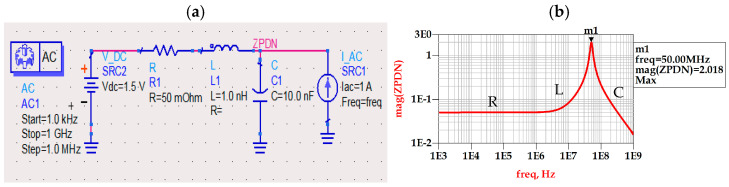
PDN frequency domain analysis. (**a**) AC PDN simulation setup. (**b**) PDN frequency domain profile showing resistive, inductive, and capacitive behavior.

An example of the frequency domain analysis of the PDN impedance is shown in Figure 3a. The PDN is modelled as an RLC circuit representing the package and PCB RL model along with the die decoupling capacitance (i.e., C). The magnitude of the impedance plot shown in Figure 3b serves to identify the PDN resonance frequency and the bandwidth as well. Basically, PDN acts as a band-pass filter to the current activity generated by the random input bit sequence.

**Figure 4:**
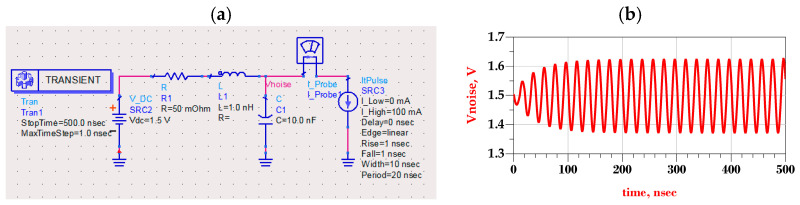
Worst-case supply ripple as IO buffer current activity period hits the PDN resonance frequency $f_{res}=\frac{1}{2\pi \sqrt{LC}}$. (**a**) Transient simulation setup. (**b**) Supply voltage time domain waveform.

This work aimed to provide improved IBIS predriver’s modelling accounting for the worst-case P/G supply variations at the predriver stage. Accordingly, the highest P/G supply amplitude variations occurs as the period of bit pattern or current activity (i.e., $i_{Hp}\left(t\right)$ and $i_{Lp}\left(t\right)$) hits the PDN resonance frequency of P/G supplies. For instance, the transient simulation setup, as shown in Figure 4a, illustrates the worst-case supply ripple time domain waveform induced by the IO buffer current activity modeled as a pulse signal with a 20 ns period (i.e., $T≅1/f_{res}$).

As seen in Figure 4b, the worst-case supply voltage waveform leading to the highest peak-to-peak jitter performance was a sinusoidal like signal. Although, the worst P/G supply waveform and frequency contents also depend on PDN characteristics such as the bandwidth and whether it presents several resonance frequencies; this paper mainly focused on developing an enhanced parametric predriver nonlinear dynamic behavior modelling for capturing the amplitude and timing distortions, as PGSV shows multi-tone sinusoidal waveforms with the highest frequency and amplitude variations leading to the worst-case jitter distortions [13,14,15,16]. Experimental measurement and simulation of power integrity test-benches show that worst-case steady-state supply ripple waveforms behave as a distorted sinusoidal voltage waveform [13,14,15,16].

Hence, the proposed modelling methodology can be used in conjunction with frequency domain approaches for PGSIJ determination as depicted by integrated frequency and time domains flow as shown in Figure 2.

This work addressed the challenge of capturing the effect of PGSV noise applied on the stages of the driver (e.g., predriver and last stage) by investigating a neural-network (NN)-based parametric model for modelling the predriver’s timing and amplitude distortions, as it is powered independently from the last-stage one. The rest of the paper is organized as follows. Section 2 details the problem formulation. Section 3 describes the proposed modelling methodology. Section 4 presents the model implementation and validation results of the proposed model’s interpolation and extrapolation under several test-case scenarios. Summary and conclusions are drawn in Section 5.

## 2. Problem Formulation

The I/O device under modelling is composed of two stages: predriver and last stage. The predriver is composed by three cascaded CMOS inverters and the last stage is represented by one inverter. The predriver is separately powered by supply voltages ($V_{dd}/V_{ss}$) from the last-stage ones $\left(V_{ddq}/V_{ssq}\right)$. Both I/O buffer stages P/G supplies are assumed to allow $\pm 10{\%\;V}_{dc}$ of ripple noise variations.

**Figure 5:**
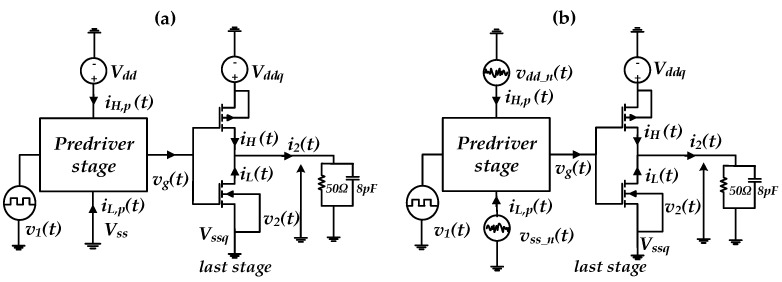
Simulation setup used to evaluate the impact of PGSV variations at predriver stage; (**a**) nominal supply case (**b**) predriver’s PGSV noise case.

**Figure 6:**
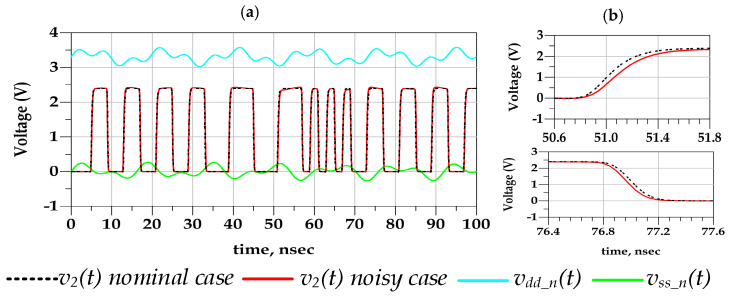
(**a**) Comparison of $v_{2}\left(t\right)$ timing waveforms in the nominal case (i.e., dc P/G supply) and predriver’s PGSV noise cases, (**b**) a zoomed version of the rising and falling edges transition.

**Figure 7:**
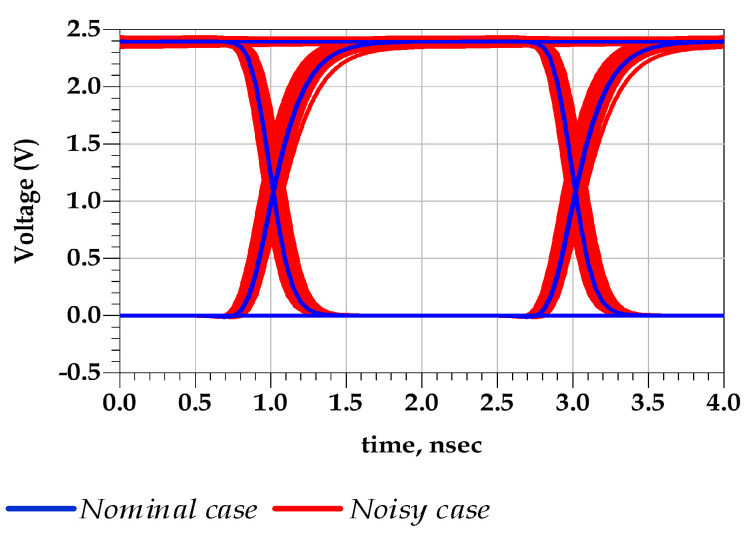
Comparison of the $v_{2}\left(t\right)$ eye diagram in the ideal supply case and predriver’s PGSV noise case.

For illustration purposes, the I/O buffer transistor level (TL) circuit was simulated under two conditions. The first scenario assumed that I/O device is powered by a nominal (fixed voltage) PGSV, as shown in Figure 5a. The second scenario simulated the case where a sum of two tones of sinusoidal voltage signal sources are only connected at the predriver’s stage PGSV terminals while last stage supplies are kept constant, as shown in Figure 5b. This analysis clearly demonstrates that the effect of timing and amplitude distortion of PGSV are induced by the predriver stage. The resulting driver output voltage, $v_{2}\left(t\right)$, under the above-described conditions is presented in Figure 6 and their respective eye diagrams are shown in Figure 7. The peak-to-peak (p2p) jitter under nominal and noisy cases are 17.15 ps and 197.519 ps, respectively. The eye height values under nominal and noisy cases are 2.38 V and 2.33 V, respectively.

The difference between the reference TL model and tow-piece IBIS-like behavioral models in predicting the output voltage timing distortion is due to the fact that the IBIS model mathematical formulation does not include the predriver’s PGSV variations and, consequently, it fails to predict the predriver’s I/O timing distortion under PGSV noise. Accordingly, the development of an improved parametric behavioral model of the active predriver’s circuit was addressed in this work based on nonlinear dynamic NN, which extends the two-piece IBIS behavioral model to also account for the predriver’s distortions under PGSV variations.

Moreover, the NN-based behavioral model enables surrogate approximation of nonlinear dynamic function with a good accuracy level. Indeed, the mathematical structure of a dynamic NN approach [5,17,18,19] has been explored in modeling a nonlinear I/O driver circuit defined by nonlinear differential equations, which is important for transient SPI analysis. For example, NN parametric models based on nonlinear system identification theory have been used to improve IBIS model for the last stage [19]. Furthermore, this modeling methodology accurately approximates the observed nonlinear dynamic memory effects from the identification electrical I/O signals without assuming a predefined equivalent circuit model template. This provides high modelling flexibility to cover a wide range of I/O buffer model design structures while disregarding the electrical physical details of the predriver or last-stage circuits. Moreover, several research works have demonstrated that the NN can yield better computational efficiency than traditional SPICE models [5,10,11,12,17,18].

## 3. Proposed Modelling Methodology

**Figure 8:**
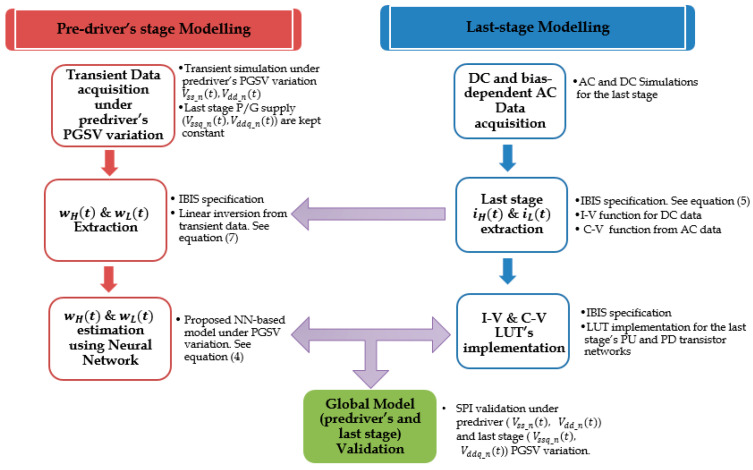
Block diagram of the I/O buffer behavioral modelling accounting for nonlinear dynamic distortion induced by the distinct P/G supplies of the predriver and last stage.

This section describes the generation of behavioral model of I/O buffer both stages under distinct PGSV variations. The block diagram of the proposed nonlinear behavioral modelling methodology of the predriver and last stage is presented in Figure 8. It shows the separate modelling steps of both drivers’ stages and the interaction between them in collecting the identification signals for training the NN model to model the predriver’s electrical behavior under PGSV variations. Accordingly, the global I/O buffer model structure is presented in Section 3.1. Section 3.2 and Section 3.3 describe the modelling methodology of the equivalent-circuit last stage model and the NN-based predriver model to accurately predict the predriver’s output STS under PGSV variations, respectively.

### 3.1. Model Structure

The standard multiport two-piece behavioral model structure, which describes the nonlinear dynamic electrical behaviors of the I/O buffer circuit, can be formulated mathematically by (2) and (3).

(2) $$\{\begin{array}{c} i_{2}\left(t\right)=\underset{k=L,H}{\sum }W_{k}\left(t\right)\cdot I_{k}\left(t\right)\\ I_{k}\left(t\right)=F_{k}\left[x_{k}\left(t\right),\frac{dx_{k}\left(t\right)}{dt}\right],\;k=L,H \end{array}\;$$

The output current, $i_{2}\left(t\right)$, is expressed as a summation of two submodels modelling the pull-up (PU) and pull-down (PD) switching activities. Each submodel is formed by multiplying the last stage current extracted at dc input stage, $I_{k}\left(t\right)$, by the switching time signal (STS), $W_{k}\left(t\right)$, capturing the I/O predriver’s timing distortions under fixed P/G supply. The PU and PD output voltage differences are defined as $x_{L}\left(t\right)=v_{2}\left(t\right)-v_{ssq\_n}\left(t\right)$ and $x_{H}\left(t\right)=v_{ddq\_n}\left(t\right)-v_{2}\left(t\right)$, respectively. They are applied to the $F_{L}\left(\cdot \right)$ and $F_{H}\left(\cdot \right)$ functions that model the nonlinear dynamic output admittances of the driver’s last stage under “*L*” and “*H*” input logic levels, respectively.

**Figure 9:**
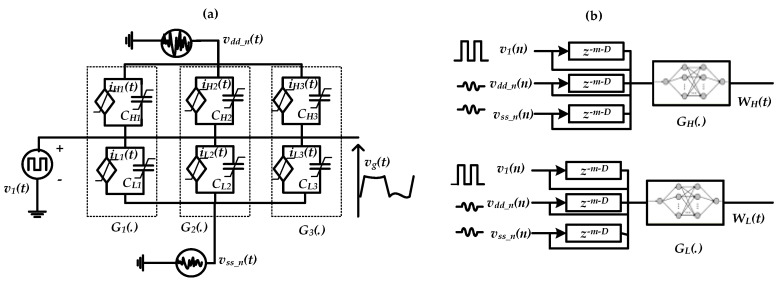
Multilayer NN-based nonlinear dynamic model representation approximating the large-signal equivalent circuit of three-stage CMOS predriver’s circuit, (**a**) the predriver equivalent circuit, (**b**) the proposed multilayer NN model for the PU and PD STS under the PGSV variations.

The large-signal equivalent circuit of the three-stage CMOS predriver’s circuit is presented in Figure 9a. It is composed of cascaded I-V and C-V functions of each CMOS inverter. The output gate voltage, $v_{g}\left(t\right)$, of predriver’s stage under PGSV variations can be formulated in continuous time domain as follows.

(3) $$v_{g}\left(t\right)=G_{1}\left(G_{2}\left(G_{3}\left(v_{1}\left(t\right),\frac{dv_{1}}{dt},v_{dd\_n}\left(t\right),\frac{dv_{dd\_n}\left(t\right)}{dt},v_{ss\_n}\left(t\right),\frac{dv_{ss\_n}\left(t\right)}{dt},\frac{dv_{g}}{dt}\right)\right)\right)\;$$

where $G_{1}\left(\cdot \right),G_{2}\left(\cdot \right),\;$and $G_{3}\left(\cdot \right)$ are multi-input single-output nonlinear functions that mathematically represent the nonlinear distortion induced by the each of the CMOS inverter stage forming the predriver’s circuit. The derivative accounts for the capacitive coupling between input, output, and power/ground supply terminals. The continuous time domain formulation can be discretized (i.e., $dx/dt≅\left(x\left(nTs\right)-x\left(\left(n-1\right)Ts\right)\right)/T_{s}$ and approximated as a direct formulation for a finite memory of the predriver’s circuit. Accordingly, Figure 9b presents the proposed multilayer NN parametric model for the PU and PD predriver’s switching activities under PGSV variations, which are also formulated in (4).

The predriver model structure relating the STS, $W_{k}\left(t\right)$, to $v_{1}\left(n\right)$, $v_{dd\_n}\left(n\right)$, and $v_{ss\_n}\left(n\right)$ that mimic the I/O timing behavior of the predriver stage.

(4) $$W_{k}\left(n\right)=G_{NN_{k}}\left(\begin{array}{c} \begin{array}{ccc} v_{1}\left(n-D\right) & \;v_{1}\left(n-D-1\right)\; & v_{1}\left(n-D-m\right), \end{array}\\ \begin{array}{ccc} v_{dd\_n}\left(n-D\right) & \;v_{dd\_n}\left(n-D-1\right) & \;v_{dd\_n}\left(n-D-m\right), \end{array}\\ \begin{array}{ccc} v_{ss\_n}\left(n-D\right)\; & v_{ss\_n}\left(n-D-1\right) & \;v_{ss\_n}\left(n-D-m\right) \end{array}, \end{array}\right)\;k=H,L\;$$

where $G_{NN}{}_{k}\left(\cdot \right)\;$is a multiple-input single output nonlinear function that maps the relationship between $W_{k}\left(t\right)\;$and the instantaneous and previous samples of the $v_{1}\left(n\right)$, $v_{dd\_n}\left(n\right)$, and $v_{ss\_n}\left(n\right)$. m represents the number of the delay steps considered for NN inputs and D represents the dead time difference determined between the output STS and the input voltage. The dead time D should be adequately identified to ensure the causality of the model.

Furthermore, NN multi-layer structure can be defined by the CMOS stage forming the predriver’s circuit. For instance, if the number of the predriver’s CMOS stage circuit is known a priori, the number of hidden layers can be determined. NN training can be an iterative process to optimize the number of hidden layers and their respective neurons while ensuring the convergence nonlinear optimization algorithm with the simplest NN structure with fewer neurons.

### 3.2. Last Stage Modelling

The last-stage model consists of summation of the conduction current modelled as current-voltage (I-V) and displacement of the current capacitance-voltage (C-V).

(5) $$F_{k}\left(t\right)=IV_{k}\left(x_{k}\left(t\right)\right)+CV_{k}\left(x_{k}\right)\frac{dx_{k}\left(t\right)}{dt},\;k=L,H$$

**Figure 10:**
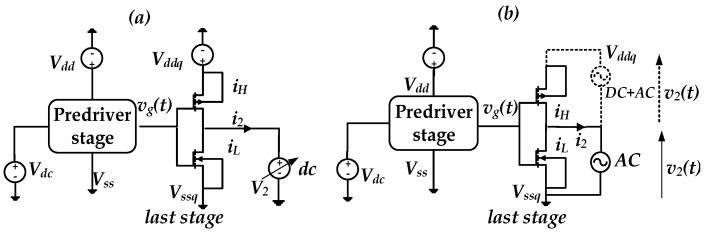
Last-stage I-V and C-V function extraction for the PU and PD devices. (**a**) DC simulation setup: I-V extraction. (**b**) AC simulation setup: C-V extraction.

This electrical model formulation, presented in (5), considers not only the static contribution of the PGSV fluctuation, but also the dynamic distortion introduced by the PU and PD capacitances, which are represented by the derivatives [5,10,20]. $IV_{k}\;\left(\cdot \right)$ functions, that capture the PU and PD transistors in the linear and the nonlinear operating ranges, were extracted by means of voltage DC sweep as shown in Figure 10. I/O buffer supply voltage for both stages were kept constant while the output voltage source was swept between$\;\left[-\cdot ,\;V_{ddq}+\cdot \right]$ for different input voltages, $v_{1}$, state, $v_{1}=0$, and then $v_{1}=V_{dd}$. The last-stage model (5) only considers the nonlinear dynamic behavior of the intrinsic effect of the active I/O buffer while the extrinsic effect of the PDN (RLC model) was reflected in the estimated supply ripple noise, as shown in Figure 3.

Furthermore, the capacitance voltage functions $s\;CV_{K}\left(\cdot \right)$ capture the dynamic distortions, which improve jitter prediction accuracy introduced by the PGSV variations. These functions were extracted via bias-dependent AC simulation at the driver’s output while the input dc voltage was kept as low or high-logic levels as illustrated in Figure 10b. The AC simulation was mainly performed in two steps to identify the power capacitor $CV_{H}\left(\cdot \right)$ and the ground capacitor $CV_{L}\left(\cdot \right).$ Firstly, the AC voltage source was connected to the last stage ground while the input $V_{dc}=0V$. Then, it was connected to the power source of the driver last stage while $V_{dc}=V_{dd}$, presented by the dashed line.

It is worth noting the perturbation assumption of the P/G voltage, where linear approximation of the I-V functions can be used because the biasing region of the PU and PD transistors of the driver’s last stage will not be severely affected. Therefore, a small-signal transistor model for P/G-induced jitter can be used by including the linear capacitive effects [19].

### 3.3. Predriver Modelling

**Figure 11:**
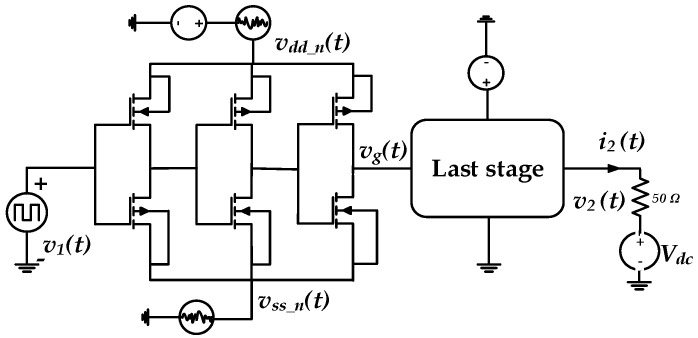
Transient simulation for the predriver STS extraction setup.

For the predriver’s model extraction setup, a transient simulation was performed in the first place. As is demonstrated in Figure 11, the input signal $v_{1}\left(t\right)$ is presented by a random bit sequence and the applied P/G supply, $v_{dd\_n}\left(t\right)$ and the $v_{ss\_n}\left(t\right)$, are defined as follows:

(6) $$\{\begin{array}{c} v_{dd\_n}\left(t\right)=V_{DC}+\underset{i}{\sum }a_{di}\;\sin\left(2\pi \cdot f_{di}\cdot t\right)\\ v_{ss\_n}\left(t\right)=\underset{i}{\sum }a_{si}\;\sin\left(2\pi \cdot f_{si}\cdot t\right) \end{array}$$

where $a_{di}$ and $a_{si}$ are the amplitudes and $f_{di}$ and $f_{si}$ are the noise frequencies. While the driver last stage supplies were kept constant to retrieve only switching identification time series signals $\{v_{1}\left(t\right),v_{dd\_n}\;\left(t\right),v_{ss\_n}\;\left(t\right),i_{2}\left(t\right),v_{2}\left(t\right)\}$ under two loading conditions (i.e., load (a) is $V_{dc}=V_{DD}$ and load (b) $V_{dc}=0V)$ that reflect the predriver’s timing distortion under PGSV variations.

To ensure a good modeling process, it is crucial to verify the coverage area of the $v_{dd\_n}\left(t\right)$ voltage variations vs. the $v_{ss\_n}\;\left(t\right)$ voltage variations. Once the driver’s last model (5) was generated, time series data recorded under two loading conditions from Figure 11 were used to determine the STS, $W_{H}\left(t\right)$, and $W_{L}\left(t\right)$ by linear inversion presented in (7):

(7) $$\left[\begin{array}{c} W_{H}\left(t\right)\\ W_{L}\left(t\right) \end{array}\right]={\left[\begin{array}{c} \begin{array}{cc} F_{L_{a}}\left(t\right) & F_{H}{}_{a}\left(t\right) \end{array}\\ \begin{array}{cc} F_{L_{b}}\left(t\right) & F_{H}{}_{b}\left(t\right) \end{array} \end{array}\right]}^{-1}\;\left[\begin{array}{c} i_{a}\left(t\right)\\ i_{b}\left(t\right) \end{array}\right]$$

where $F_{L_{a}}$, $F_{H_{a}}$, and $i_{a}$ are the extracted data corresponding to the load (a) and $F_{L_{b}}$, $F_{H_{b}}$, and $i_{b}$ correspond to the load (b).

After causing the STS to reflect the predriver’s distortions under PGSV variations, NN-model’s parameters or coefficients were identified based on non nonlinear optimization back-propagation algorithm (i.e., Levenberg-Marquart) [5,6,17].

## 4. Model Implementation and Validation Results

The proposed modelling framework was validated with extracted data from I/O buffer TL circuit dc, ac, and transient simulations. Two I/O buffer’s technologies and topologies were considered in this validation. For the predriver’s model validation, a 0.35 μm TSMC CMOS multistage I/O buffer was considered to perform model’s extraction and validation. In this case, last stag’s PGSV are kept constant; therefore, only the PSIJ from the predriver is considered. Additionally, I/O buffer circuit with slew rate control based on fully depleted silicon on insulator (FDSOI) 28-nm technology was used to extract behavioral models and validate the global model performance under PSIJ from both predriver’s and last-stage electrical circuits.

**Figure 12:**
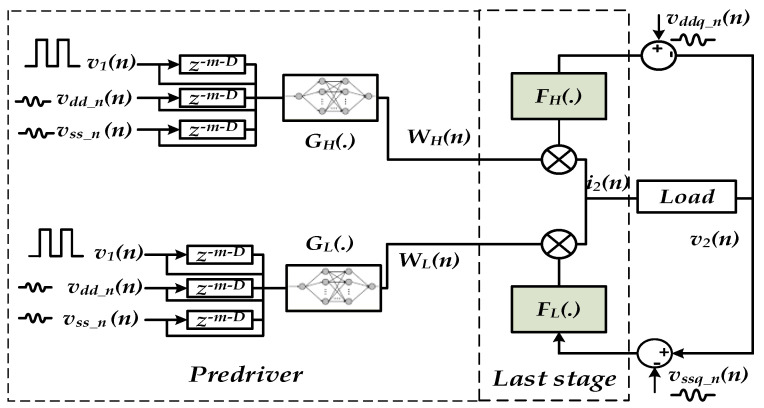
I/O buffer implementation in Simulink considering PGSV variations applied on the predriver and on the last stage separately.

Look-up tables (LUTs) were used to implement the last-stage PU and PD, I-V and C-V functions. Extracted coefficient of the NN-based parametric model using hyperbolic tangent activation functions was implemented in the MATLAB Simulink time-domain solver tool as shown in Figure 12. Two NN-based parametric submodel structures, $G_{NN}{}_{k}\left(\cdot \right)$, were trained to extract the coefficient (e.g., parameters) of the multilayer NN algorithm. The NN structure is mainly composed by two hidden layers with four neurons in each layer. The different parameters used for the NN-based model construction is presented in Table 1.

During the identification stage of the NN-based parametric model, a different number of hidden layers and a different number of neurons per layer were tested in order to ensure better tradeoff between model’s complexity and accuracy. Moreover, to evaluate the model accuracy and performance, different validation setups were performed and are detailed in the next subsections.

### 4.1. Predriver Model Validation

**Figure 13:**
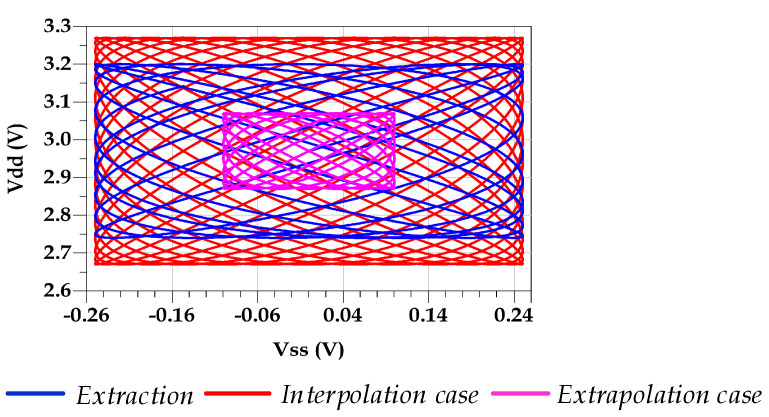
Coverage area of $v_{dd\_n}\left(t\right)$ vs. $v_{ss\_n}\left(t\right)$ for the extraction setup, interpolation case, and extrapolation case.

The first validation setup consists of evaluating the performance of the proposed driver’s modelling. Therefore, we carried out a comparative study between the extracted $W_{k}\left(t\right)$ from TL circuit V-t data and the estimated one using the current modeling methodology in two different conditions. Two test cases of validation data were used to evaluate the interpolation and extrapolation capabilities of the extracted model, and Figure 13 illustrates the coverage area of the $v_{dd\_n}\left(t\right)$ vs. $v_{ss\_n}\left(t\right)$ data used in the extraction along with both interpolation and extrapolation test cases. Table 2 presents the used data in the two different validation scenarios.

**Figure 14:**
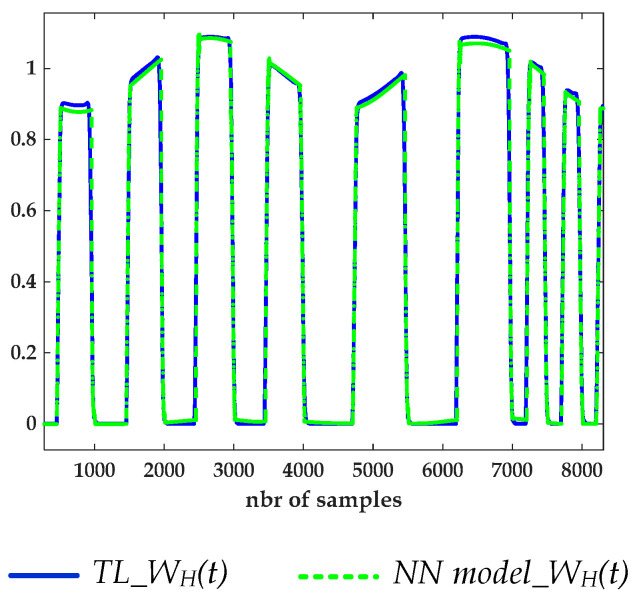
Comparison between the extracted $W_{H}\left(t\right)$ STS from TL V-t data under PGSV variations and the estimated STS using the NN model.

**Test case 1:** The PGSV’s amplitudes which were applied to the predriver terminals were lower than the data used during the extraction setup. In this interpolation scenario, the extracted STS (e.g., $W_{H}\left(t\right)$) from the TL-circuit-simulated data and the predicted signal by the proposed parametric NN-based model are compared in Figure 14. It is noticeable that the predicted $W_{H}\left(t\right)$ waveform mimics the reference STS, which is determined from the TL V-t data extracted under PGSV variations, during the rising and falling transitions, as well as in the amplitude distortion.

**Figure 15:**
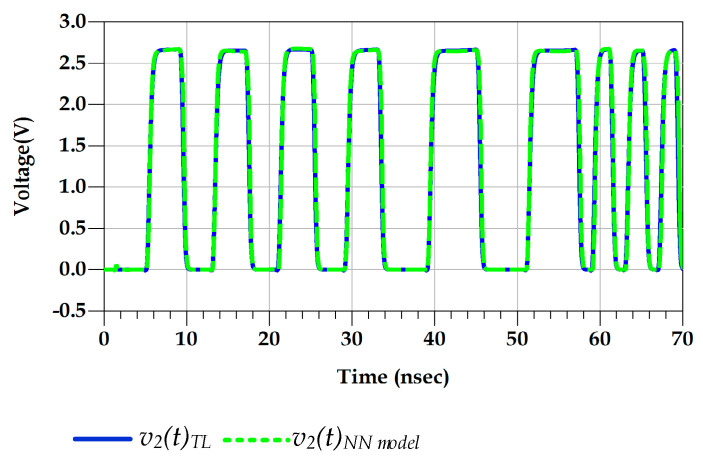
Comparison of the $v_{2}\;\left(t\right)$ waveform of the TL circuit and NN models under predriver’s PGSV variations (test case 1).

**Figure 16:**
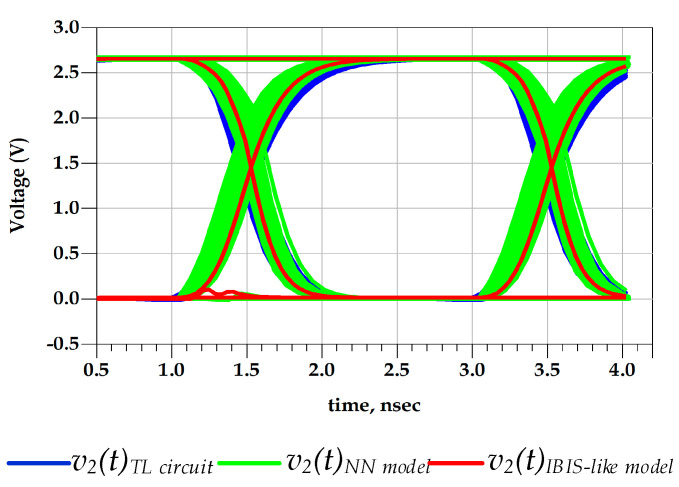
Comparison of $v_{2}\left(t\right)$ eye diagrams under predriver’s PGSV variations (test case 1).

Figure 15 shows the good agreement between the predicted output voltage by the reference TL circuit and the proposed behavioral models. Consequently, Figure 16 demonstrates that the eye diagram of the proposed model perfectly mimics the TL output eye diagram while the output eye diagram of the IBIS-like model fails.

The eye-opening measurements were performed under 40–60% eye boundary, and the eye threshold levels were set as 20% to 80% points on the rising and falling transitions. In fact, the timing distortion induced by the predriver PGSV variations is not captured by IBIS model because V-t data are extracted at fixed PGSV. These observations are confirmed by the numerical value of the eye diagram metrics reported in Table 3. A difference of 8.9 ps between the p2p jitter of the proposed model and the reference TL circuit model was observed.

Therefore, the relative error of the p2p eye’s jitter is 4.3% and 48%, shown by the proposed model and the IBIS-like model, respectively. The eye height is almost the same in the three eye diagrams. Consequently, predriver’s circuit induces, mainly, timing distortions at the last’s stage output voltage.

**Figure 17:**
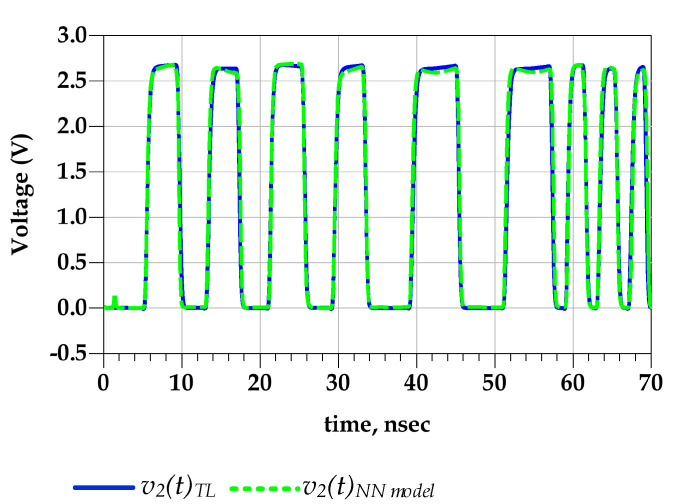
Comparison of $v_{2}\;\left(t\right)$ waveform of the TL circuit and NN models under predriver’s PGSV variations (test case 2).

**Figure 18:**
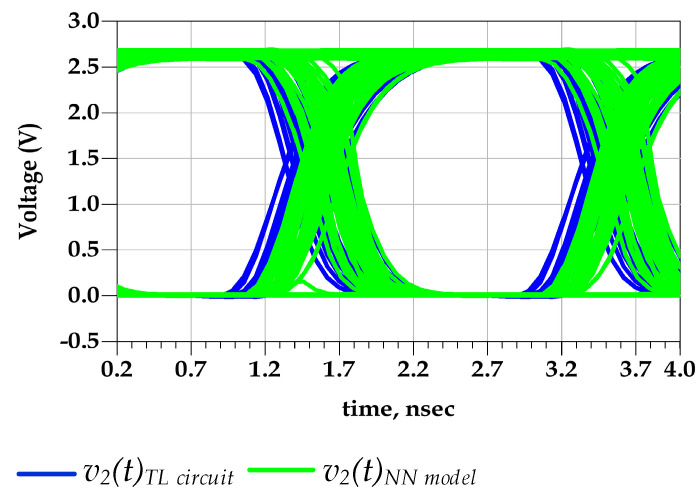
Comparison of eye diagrams of $v_{2}\left(t\right)$ under predriver’s PGSV variations (test case 2).

**Test case 2:** This validation setup assesses the extrapolation capabilities of the behavioral model. In fact, the PGSV amplitudes applied to the predriver terminals exceeds the amplitude of signals used as excitation during the extraction setup. Figure 17 shows a good match between the predicted output voltage from the proposed behavioral and the reference TL circuit models. The prediction accuracies of the eye openings are depicted in Figure 18 and their metrics are summarized in Table 4. The difference between the TL reference circuit and the NN model in the extrapolation condition of p2p jitter is 45.59 ps, which is about 9.9%. The eye height of the TL circuit and the NN model are 2.54 V and 2.53 V, respectively.

To conclude, the results of these validation setups prove that the proposed parametric NN model presents a good accuracy level in the interpolation and extrapolation conditions.

### 4.2. Global Model Validation under PGSV Variations at the Predriver and Last-Stage

To ensure the model stability and reliability, a second validation step, illustrated in Figure 18, was performed. Two NN structures were used to estimate the predriver’s nonlinear memory behavior. In the current simulation, decoupled P/G supply noise sources were applied at both predriver’s and last-stage terminals.

**Test case 3:** sinusoidal PGSV sources were applied at the last stage $v_{ddq\_n}\left(t\right)=V_{ddq}+a_{dl}\;\sin\left(2\pi \cdot f_{dl}\cdot t\right)$ and $v_{ssq\_n}\left(t\right)=a_{sl}\;\sin\left(2\pi \cdot f_{sl}\cdot t\right)$ with the following parameters: $a_{d_{l}}=0.1\;V$, $f_{d_{l}}=70\;\mathrm{MHz}$ and $a_{s_{l}}=0.2\;V$, $f_{s_{l}}=75\;\mathrm{MHz}$. The amplitudes and the frequencies of the P/G sinusoidal sources applied at the predriver stage were $a_{d}=0.12\;V$, $f_{d}=90\;\mathrm{MHz}$ and $a_{s}=0.1\;V$, $f_{s}=80\;\mathrm{MHz}$.

**Figure 19:**
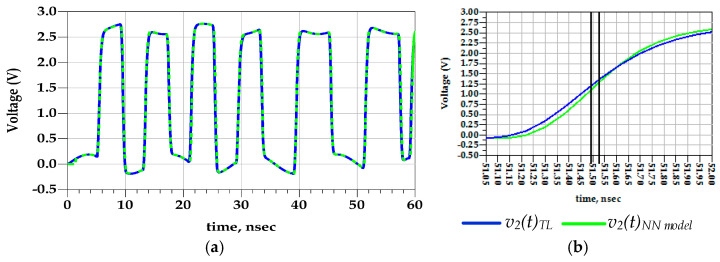
(**a**) Comparison of $v_{2}\left(t\right)$ waveform of the TL circuit and NN models under distinct PGSV variations applied at both driver’s stages, (**b**) a zoomed version of the rising transition (test case 3).

**Figure 20:**
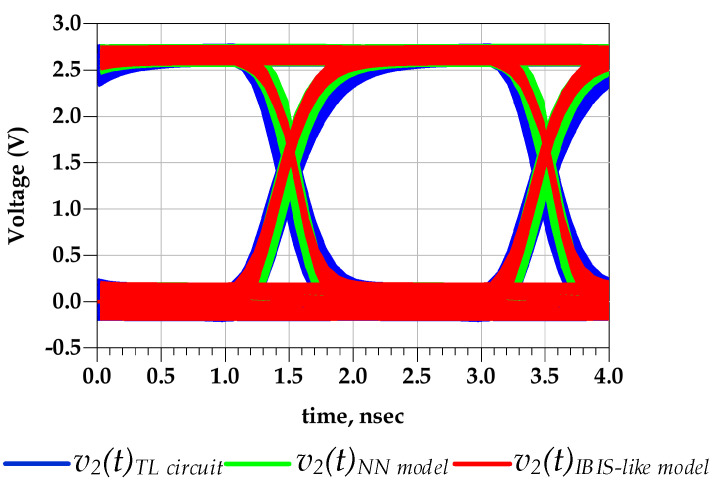
Comparison of the eye diagrams of $v_{2}\left(t\right)$ under distinct PGSV variations applied to both driver’s stages (test case 3).

Figure 19a shows the output voltage waveform prediction of CMOS 0.35 um I/O buffer TL circuit and the NN models, of I/O buffer under distinct P/G supply noise applied to both diver’s stages. Moreover, Figure 19b presents a zoomed version of the rising edge transitions. For instance, at 1.25 V, the corresponding timing of the $v_{2}\left(t\right)$ TL circuit and the NN models were 217.522 ps and 217.550 ps, respectively. These results are also confirmed by the eye diagrams plot in Figure 20 and the respective numerical results are reported in Table 5. The p2p jitter value difference between TL and proposed models was 26 ps, corresponding to 9.82% of relative error. Moreover, the difference of the p2p jitter value between the IBIS-like and the TL models was about 51.2 ps, corresponding to 23.3%.

**Test case 4:** The proposed modelling was validated considering a FDSOI 28 nm CMOS driver. A new extraction setup and NN model trainings were executed. The P/G supply noise sources of the predriver were assumed to be a superposition of two sinusoidal signals in order to evaluate the noise in a realistic scenario. Consequently, the used PGSV values presented as follows: $V_{dc}=1.5\;V$, $a_{d_{1}}=0.11\;V$, $f_{d_{1}}=125\;\mathrm{MHz}$, $a_{d_{2}}=0.03\;V$, $f_{d_{1}}=85\;\mathrm{MHz}$ and $a_{s_{1}}=0.1\;V$, $f_{s_{1}}=225\;\mathrm{MHz}$, $a_{s_{2}}=0.04\;V$, $f_{s_{2}}=160\;\mathrm{MHz}$. The P/G supply noise sources applied at the buffer last stage are: $V_{dc}=1.5\;V$ $a_{dl}=0.1\;V$, $f_{dl}=210\;\mathrm{MHz}$ and $a_{sl}=0.08\;V$, $f_{sl}=85\;\mathrm{MHz}$.

**Figure 21:**
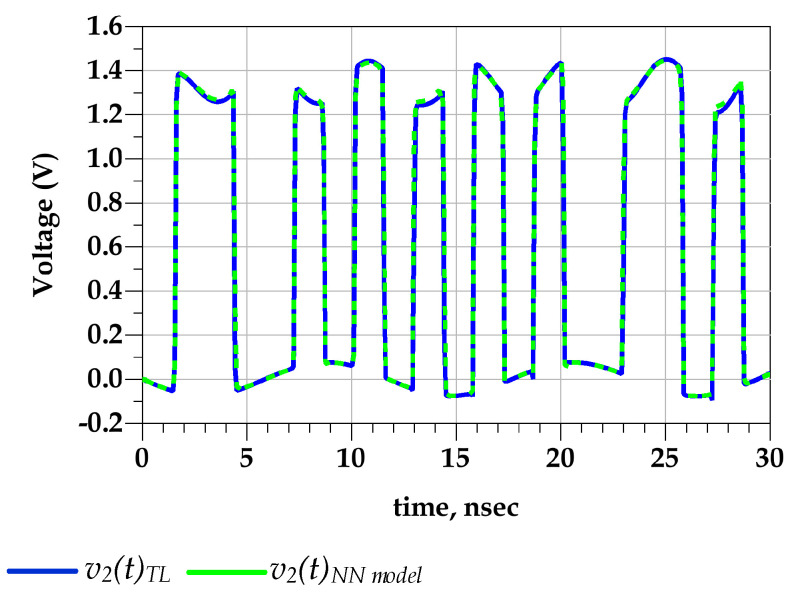
Comparison of $v_{2}\left(t\right)$ waveform of TL circuit and NN models under two-tones PGSV variations applied at both driver’s stages, for FDSOI technology (of test case 4).

**Figure 22:**
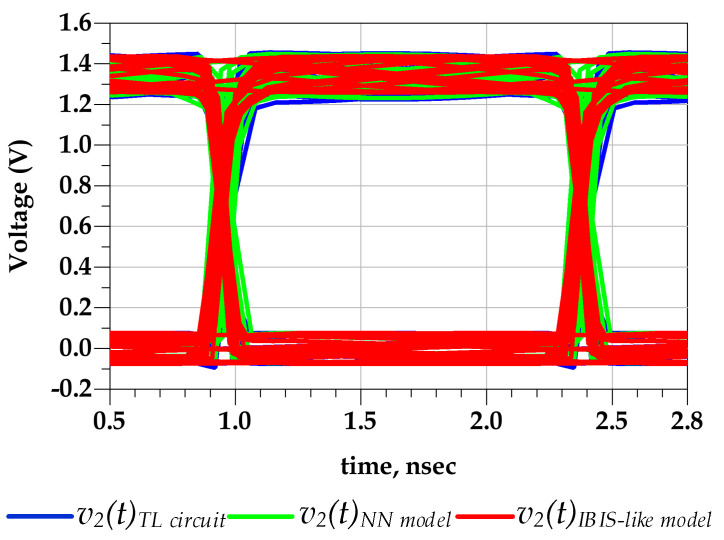
Comparison of eye diagrams of $v_{2}\left(t\right)$ under two-tone PGSV variations applied at both driver’s stages, for FDSOI technology (test case 4).

Figure 21 shows the comparison of the predicted output voltage waveforms simulated based on the TL circuit and the NN models. Besides, Figure 22 shows the eye diagrams as PGSVs were applied to predriver and last-stage terminals of the TL circuit, the NN model, and the IBIS-like model. The proposed NN-based model captures the PSIJ from both I/O buffer stages while presenting a difference of 6.2 ps that corresponds to 7.3% of relative error. However, the IBIS-like mode shows a p2p eye jitter of 39.21 ps, corresponding to 46.33% as reported in Table 6.

It is worth noting that validation with pure sinusoidal or distorted sinusoidal (i.e., two-tone) PGSV variations does not affect the predicted waveform under PGSV variations because the model was trained with multi-tone sinusoidal voltages that cover the possible frequency of interest within the bandwidth of the PDN.

## 5. Conclusions

This paper presents an improved nonlinear dynamic I/O buffer circuit behavioral modelling methodology to accurately predict the timing distortions induced by the predriver as well as by the last stage of the driver. The NN-based parametric model was developed to estimate the output switching time signals of the predriver under the power ground supply variations. The proposed model demonstrates good results in estimating the PSIJ with a decoupled supply source noise at the predriver and at the last stage of the driver.

Moreover, to evaluate the proposed model’s performance in predicting the eye diagram opening and p2p jitter from transient simulation, two different I/O buffer circuit technologies were tested: 0.35 μm and 28 nm FD-SOI technologies. The simulation results of the established model showed a good approximation for the p2p eye jitter value with worst-case relative error about 9.82%

## Figures and Tables

**Table 1:** NN-based model parameters.

Parameters	Values
Ts: sampling time (ps)	8
m (ps)	3.Ts
D (ps)	150.Ts
Training epochs	200

**Table 2:** PGSV parameters used to validate the proposed model under interpolation (test case 1) and extrapolation (test case 2).

Parameters	Test Case 1	Test Case 2
$a_{d1}$ (V)	0.1	0.3
$f_{d1}$ (MHz)	90	75
$a_{s1}$ (V)	0.1	0.25
$f_{s1}$ (MHz)	80	80

**Table 3:** Jitter performance of the TL circuit, IBIS-like, and NN models under predriver’s PGSV variations (test case 1).

	TL Circuit	NN Model	IBIS-Like Model
Eye jitter (p2p) (ps)	203.99	212.86	35.48
Eye width (ps)	1835.92	1898.01	1995.56
Eye height (V)	2.58	2.59	2.62

**Table 4:** Jitter performance of the TL circuit and proposed models under predriver’s PGSV variations (test case 2).

	TL Circuit	NN Model
Eye jitter (p2p) (ps)	461.19	415.60
Eye width (ps)	1543.23	1617.23
Eye height (V)	2.54	2.53

**Table 5:** Jitter performance of the TL circuit, IBIS-like, and NN models under distinct PGSV variations applied at both driver’s stages (test case 3).

	TL Circuit	NN Model	IBIS-Like
Eye jitter (p2p) (ps)	219.72	198.12	168.51
Eye width (ps)	1809.31	1862.53	1942.35
Eye height (V)	2.31	2.34	2.38

**Table 6:** Jitter performance of TL circuit, IBIS-like, and NN models under two-tone PGSV variations applied at both driver’s stages (test case 4).

	TL Circuit	NN Model	IBIS-Like
Eye jitter (p2p) (ps)	84.62	90.82	45.41
Eye width (ps)	1341.04	1336.35	1381.77
Eye height (V)	1.15	1.16	1.182
